# Early Bowel Lengthening Procedures: Bi-Institutional Experience and Review of the Literature

**DOI:** 10.3390/children9020221

**Published:** 2022-02-07

**Authors:** Elisa Negri, Riccardo Coletta, Lynette Forsythe, Francesca Gigola, Maria Chiara Cianci, Antonino Morabito

**Affiliations:** 1 Department of Neuroscience, Psychology, Drug Research and Child Health (NEUROFARBA), University of Florence, 50121 Florence, Italy; francesca.gigola@unifi.it (F.G.); mariachiara.cianci@unifi.it (M.C.C.); antonino.morabito@unifi.it (A.M.); 2 Department of Pediatric Surgery, Meyer Children’s Hospital, University of Florence, 50139 Florence, Italy; riccardo.coletta@meyer.it; 3 School of Environment and Life Science, University of Salford, Salford M5 4NT, UK; 4 Royal Manchester Children’s Hospital, Manchester University NHS Foundation Trust, Manchester M13 9WL, UK; lynette.forsythe@mft.nhs.uk

**Keywords:** early bowel lengthening, short bowel syndrome, autologous intestinal reconstructive surgery

## Abstract

Early bowel lengthening procedure (EBLP) has been defined as any bowel lengthening procedure performed before six months of age. The purpose of this paper is to compare our experience with literature on this subject to identify common indications. A bi-institutional retrospective analysis was performed. Diagnosis, type of surgery, age at procedure and outcomes were analysed. Eleven EBLP were performed in Manchester and Florence from 2006 to 2021. The median age at surgery was 126 days (102–180), pre-operative median short bowel (SB) length was 28 cm (17–49) with a post-operative median increase of 81%. Furthermore, a PubMed/Embase search was undertaken regarding bowel lengthening procedures performed in the last 40 years. Sixty-one EBLP were identified. The median age was 60 days (1–90). Serial transverse enteroplasty (STEP) was the most frequent procedure used, with a median increased bowel length of 57%. This study confirms that no clear consensus on indication or timing to perform early SB lengthening is reported. According to the gathered data, EBLP should be considered only in cases of actual necessity and performed in a qualified intestinal failure centre.

## 1. Introduction

Short bowel syndrome (SBS) is a severe multi-systemic disorder resulting from losing a significant amount of small bowel. The incidence of severe SBS is estimated to be 25/100,000 live births; most sufferers are infants and young children [1].

The debate about the correct definition of SBS is still open. Bowel length of less than half expected for gestational age is considered abnormal [2]. According to different authors, generally <40 cm requires therapy [3]. The most frequent causes of SBS are necrotizing enterocolitis (NEC), small intestinal volvulus in intestinal malrotation, gastroschisis and small bowel atresia. In SBS, the residual intestine is inadequate to permit absorption/digestion of nutrients to maintain body weight, transit time is shortened, absorption of nutrients becomes ineffective and malnutrition, dehydration and electrolyte deficiency can develop [4]. In this condition, the bowel adapts, crypts deepen, and villi become hypertrophic through a slow process that usually results in massive dilatation of the bowel. In dilated intestines, muscle constrictions are ineffective and the peristalsis becomes insufficient, and stationary bowel leads to translocation of bacteria and sepsis [5].

The management of SBS has evolved tremendously over the past two decades, improving patients’ survival rates. This has mainly been due to surgical techniques and improvements in total parenteral nutrition (TPN). However, complications of TPN still represent a major challenge to paediatric intestinal rehabilitation teams [6]. Outcomes are influenced by different factors, including the site of the small bowel resected, quality of residual bowel, presence of the ileocecal valve, length of remaining colon and the presence of potential for intestinal continuity. Initial results published by Bianchi showed a survival of approximately 45% [7]. In more recent works, an improved survival rate of 92% has been reported, with 58–96% of TPN weaning [2,8].

Different bowel lengthening techniques have been used in recent years, often causing much controversy with varying results. The type of surgery is case specific and the remaining bowel length and degree of bowel distension are important factors to consider when choosing the best procedure for the patient. In case of un-dilated bowel, there can be a period of 20–24 weeks before performing an AGIR procedure, in which a tube stoma system can be applied to allow bowel expansion. [2,5] Oral nutrition is generally preferred to stimulate the swallowing mechanism and to avoid food aversion during this period [9].

The most frequently chosen autologous gastrointestinal reconstructions (AGIR) are longitudinal intestinal lengthening and tailoring (LILT), proposed by Bianchi in 1980 [10], and serial transverse enteroplasty (STEP), proposed by Kim in 2003 [11]. Both procedures provide a better outcome if intestinal dilatation has occurred. More recently, the spiral intestinal lengthening and tailoring technique (SILT) has been introduced, which allows lengthening of an intestinal segment with a lesser degree of bowel dilatation [12]. Other techniques, such as anti-peristaltic reverse segment or the more recently introduced transverse flap duodenoplasty, can be applied in combination with older techniques [13].

Despite there being no consensus about the correct timing to perform a lengthening procedure, some years ago it was suggested that early surgical intervention took advantage of normal growth and development [6]. Some authors suggested a benefit of earlier adaptation when AGIR surgery is offered during the first 365 days of life [14], while others recommended surgical intervention only after a period of bowel adaptation [15].

Several studies have demonstrated that a multidisciplinary approach to SBS dramatically improves the surgical and medical outcomes of these patients [16,17]. Modern intestinal rehabilitation centres can offer these patients the most updated options to achieve the best possible results such as an unstructured pathway that includes medical, surgical and psychological support [6] tailored to individual patient needs [18,19].

A structured pathway for managing these patients was proposed by the Manchester Children’s Hospital team in 2012 [6]. However, the timing of intervention in patients with short bowel syndrome is still debated.

The purpose of this review about early small bowel lengthening (elongation procedures performed before six months of life) is to examine outcomes in combination with our experience.

## 2. Materials and Methods

Children with short bowel syndrome treated at Royal Manchester Children’s Hospital and at Meyer Children’s Hospital of Florence between 2006 and 2021 were retrospectively analysed by personal reference and review of medical records.

A comprehensive literature review was conducted in November 2021 in accordance with the 2020 Preferred Reported Items for Systematic reviews and Meta-Analysis (PRISMA) [20]. Two different reviewers independently extracted data, with discrepancies resolved by consensus. A PubMed/Embase search for all the children who underwent bowel lengthening procedures before 6 months of age was performed. Research terms were: short bowel syndrome, intestine, lengthening, neonatal, and children. Reviews and papers not in English were excluded from this investigation. Data were recorded in a Microsoft Excel dataset in chronological order.

We analysed papers’ characteristics: type of article, number of cases described, number of early procedures; and patient’s characteristics: primary diagnosis, age at procedure, reason for early lengthening, small bowel length before and after surgery, type of procedure, number of cases in which parenteral nutrition was stopped and its duration, number of deceased patients, and complications after surgery.

Numerical data are presented as median ± interquartile range. This study was conducted in accordance with the International Conference for Harmonization Good Clinical Practice (ICH-GCP) and with the current revision of the Declaration of Helsinki.

## 3. Results

From 2006 to 2021, 11 EBLP were performed by the same surgeon at the Royal Manchester Children’s Hospital and at Meyer Children’s Hospital of Florence (out of a total of lengthening procedures); five were males and six females. Median gestation at birth (weeks) was 34 (32.5–34), and median age at surgery was 126 days (102–180). The primary diagnoses were six gastroschisis, three NEC, one intestinal atresia with volvulus and one multiple atresia.

Indications for early lengthening were re-dilatation following primary anastomosis, failure of enteral nutrition, and allowing for natural SB growth. Survival rate was 90.9%; none of the patients required repeated lengthening. Four reached full enteral autonomy and four partial home parenteral nutrition. The breakdown of the type of lengthening procedures performed at the hospital were: three LILT, three STEP, three SILT, one LILT and STEP, and one STEP and reverse interposition. Pre-operative median SB length was 28 cm (17–49), while post-operative median SB length was 51 cm (25–70), with a median increased bowel length of 81%.

**Figure 1:**
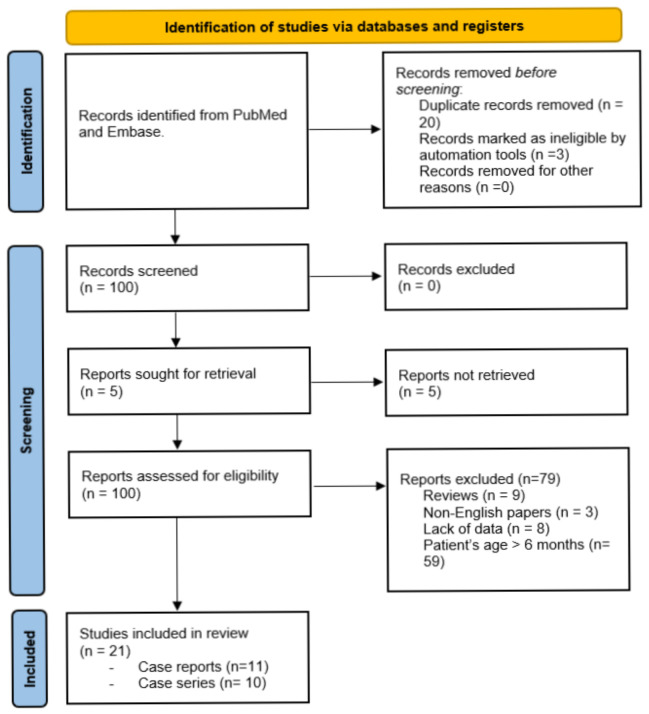
PRISMA flow diagram [20].

Searching the literature, 100 papers were reviewed to reveal more than 400 lengthening procedures performed over a 40-year period. Twenty-one papers matched our criteria, including 10 single centre studies and 11 case reports. Sixty-one early short bowel lengthening procedures were recorded (Figure 1).

The AGIR procedures reported were: STEP (36), LILT (20), anterior flap (4) and duodenal flap (1). The main reason for the early lengthening procedure was excessive dilatation due to SB atresia (22 cases), followed by failure of enteral feeding (3), failure of enteral feeding with Sepsis (1), failure of enteral feeding with bowel dilatation (1), high stool output (5) and a very short bowel (1). In the remaining cases, it was not possible to determine the clinical indication to perform an EBLP from the article (N/A).

Median age at lengthening was 60 days (1–90). Median initial SB length was 40 cm (20–62.5), and median length achieved was 63 cm (47–85), with a median increased bowel length of 57%. Parenteral nutrition was stopped after a median time of 180 days (90-247.5). Fifteen patients (25%) weaned off PN (Table 1).

Post operative complications reported were sepsis (19), re-STEP procedure due to excessive dilatation (15), intestinal failure-associated liver disease (IFALD) (3), bowel obstruction (3), enterocutaneous fistulas (2), cholestasis (1), low body weight after procedure (1), anastomosis leakage (1), and transaminase elevation (1). Eight patients underwent intestinal transplantation, while one required liver transplantation. Overall, 25% of patients died due to liver failure (9), unspecified “SBS complications” (2), sepsis with liver failure (1), sepsis (1), heart disease (1) and upper gastrointestinal haemorrhage (1). Papers and patients’ characteristics are summarized in Table 2.

## 4. Discussion

Current management of children with short bowel syndrome is known to be a complex, prolonged process where the paediatric surgeon is constantly facing difficult challenges to overcome all the nutritional and social problems related to this condition.

Our data show that the early intestinal lengthening procedures seem to have high rates of post-operative complications and re-intervention, with less-than-optimal rates of intestinal stretch compared to our experience.

Moreover, more patients needed parenteral nutrition than recent analyses, with a similar PN duration [2,18,40,41,42]. In fact, only fifteen patients (25%) weaned off PN and achieved enteral autonomy. In those patients who weaned off PN, the time to achieve enteral autonomy varied widely (30–900 days), with a duration of PN comparable to that of the same of the studies [2,40].

We have confirmed that LILT and STEP procedures are the most popular surgical techniques used for AGIR, while they may be perceived as technically challenging and they are not the best option in mildly dilated small bowel (2–5 cm). Interestingly, our study confirms that LILT and STEP successfully elongated the bowel up to 100 and 75%, respectively [3].

Initially, LILT procedures were mainly carried out by the Manchester team. The introduction of other techniques such as STEP subsequently allowed a broadening of the therapeutic options and to the possible combination of different surgical procedures. The advent of new techniques such as SILT and duodenal flap will probably contribute to further modifying these percentages and outcomes. Furthermore, we observed that patients who underwent surgery in Manchester/Florence were older and had better outcomes, probably due to both the timing of intervention and to greater team experience in managing these patients.

While some years ago early structured intervention appeared to improve the clinical outcome of infants with SBS by reducing the morbidity and mortality associated with PN [14], our data seems to be in agreement with some authors who recommended surgical intervention only after a period of bowel adaptation, as the natural process may obviate the need for any other interventions [43]. We know, in fact, that outcomes mostly depend on the ability of the residual gastrointestinal tract to adapt functionally [17].

This literature review revealed that a high rate of re-STEP procedures and sepsis episodes occur in the post-operative period. This can be explained by an excessive rapid bowel dilatation with consequent bacterial overgrowth. This involves a subsequent, second surgery in the post-operative period, with high anaesthesiologic and surgical risks for the newborn child, and does not allow for a gradual bowel adaptation, one of the main goals in SBS patients.

However, our study has some limitations. The literature review was limited by the great variability in the availability of data reported in different articles selected. We could speculate that the elevated number of deaths reported could be reflective of unreported co-morbidities as well as possible lacks of clinical data. Moreover, the study retrospectively compares an historical cohort of patients, with a group of children who underwent surgery in more recent times in specialized centres (Table 1).

## 5. Conclusions

In conclusion, SBS patients are difficult to treat from the first days of life. While previously there was the belief that we had to intervene aggressively as quickly as possible to allow better intestinal adaptation, our data report a high rate of complications and a difficulty to wean off PN. Previously, the main indication for intervention was the impossibility of feeding these patients enterally, but progress in the production of specific parenteral formulations has made it possible to reduce surgical procedures. Therefore, we believe that early lengthening procedures should be carefully considered and offered only in selected circumstances. We believe that excessive intestinal dilatation associated with severe obstructive symptoms and recurrent episodes of sepsis could be one of these.

A multidisciplinary team (MDT) with gastroenterologists, paediatric surgeons, nutritionists, radiologists, anaesthesiologists and other figures, remains of fundamental importance in improving the management and short- and long-term results of these patients. The MDT approach will clearly help patients’ selection and overall outcomes.

## Figures and Tables

**Table 1:** Literature review and personal experience.

	Literature Review	Personal Experience
Age at surgery (days; IQR)	60 (1–90)	126 (102–180)
Pre-operative SB length (cm)	40 (29–62.5)	28 (17–49)
Post-Operative SB length (cm)	63 (49–85)	51 (25–70)
% Increased length	57%	81%
Procedures		
STEP	35	3
LILT	20	3
SILT	0	3
LILT & STEP	0	1
STEP & Reverse	0	1
Anterior Flap	4	0
Duodenal Flap	1	0

**Table 2:** Results of literature review. Papers and patients’ characteristics.

		N° EBLP	Diagnosis	Indication	Mean Age (days)	Pre-OP SB (cm)	Technique	Post-OP SB (cm)	Stop PN (Time in Days)	N° of Death (Cause)	Complications (n°)
1	[21]	1	Gastroschisis and SBA	FEF	33	28	LILT	56	1 (180)		Anastomotic leakage (1)
2	[22]	1	SBA and MV	Very SB	1	12	LILT	21		1 (UGIH)	
3	[23]	1	SBA	N/A	90	10	LILT	15		1 (Sepsis)	
4	[24]	1	SBA	FEF	90	48	LILT	58	1 (180)		
5	[25]	1	MV	FEF and Dilatation	90	15	LILT	NA	1(900)		
6	[26]	14	SBA, Gastroschisis, MV, NEC	N/A	N/A	N/A	LILT	N/A	1	7 (LF)	Sepsis (14)
											IT (5)
7	[27]	1	Gastroschisis and SBA	Dilatation	1	22	STEP	51	N/A		
8	[28]	4	SBA	N/A	18	90	STEP (4)	67	N/A	Sepsis and LF (2)	IT (7)
						60		128			
						106		118		Heart disease (1)	Bowel obstruction (2)
						150		198			
9	[29]	8	SBA (2),	High stool output	88 (mean)	67 (mean)	STEP	NA	1(60)	2 (SBS complications)	IT (1)
			Internal hernia (1)								Enterocutaneous fistula (1)
			MV(3)								
			Intestinal ischemia (1)								
			Gastroschisis (1)								
10	[16]	1	Gastroschisis and SBA	FEF and septic episodes	150	40	LILT	80	1 (270)		Sepsis and bowel stenosis (1)
11	[30]	1	SBA	Dilatation	60	30	STEP	48			Feeding intolerance, sepsis and re/dilatation (1)
12	[31]	1	Gastroschisis and SBA	FEF	120	42,5	STEP (3 times)	112	1 (>5 years)		Feeding intolerance, bowel obstruction, re-dilatation and IT at 5 years old (1)
13	[32]	1	SBA	NA	3	15	STEP (2 times)	20	1 (N/A)		Grow and Vit D deficiency (1)
14	[33]	1	SBA		1	35	STEP (2 times)	50	1		
15	[34]	15	SBA (9)	Dilatation	1	32 (mean)	STEP (15) (9 re-STEP)	47 (mean)	3 (N/A)	1 (IFALD)	IFALD (2), ITL(1)
			Gastroschisis (5)								
			MV(1)								
16	[35]	1	SBA	Dilatation	12	N/A	STEP	N/A	N/A		
17	[36]	1	MV	NA	180	35	Duodenal STEP	90	1 (750)		
18	[37]	4	SBA	Dilatation	2	50	Anterior flap	60	4 (< 30 days)		Cholestasis (1)
						55		63			
						65		75			
						60		68			
19	[38]	1	Gastroschisis and SBA	Dilatation	21	90	STEP	100	1 (30)		Enterocutaneous fistula (1)
20	[13]	1	SBA	Dilatation and slow transit	60	9	Duodenal Flap	12			
21	[39]	1	Gastroschisis and SBA	N/A	180	20	STEP	N/A			

EBLP (Early bowel lengthening procedures); FEF (Failure of enteral feeding); SBA (Short bowel atresia); MV (midgut volvulus), LILT (longitudinal intestinal lengthening), STEP (serial transverse enteroplasty); UGIH (Upper gastro intestinal haemorrhage); SB (short bowel); LF (liver failure); IT (intestinal transplantation; IFALD (intestinal failure associated liver disease); ITL (intestinal and liver transplantation).

## Data Availability

The data that support the findings of this study are available on request from the corresponding author [E.N].
